# Auditory and cross-modal attentional bias toward positive natural sounds: Behavioral and ERP evidence

**DOI:** 10.3389/fnhum.2022.949655

**Published:** 2022-07-29

**Authors:** Yanmei Wang, Zhenwei Tang, Xiaoxuan Zhang, Libing Yang

**Affiliations:** ^1^Shanghai Key Laboratory of Mental Health and Psychological Crisis Intervention, School of Psychology and Cognitive Science, East China Normal University, Shanghai, China; ^2^Shanghai Changning Mental Health Center, Shanghai, China

**Keywords:** attentional bias, positive natural sounds, emotional cueing task, attention enhancement, event-related potentials

## Abstract

Recently, researchers have expanded the investigation into attentional biases toward positive stimuli; however, few studies have examined attentional biases toward positive auditory information. In three experiments, the present study employed an emotional spatial cueing task using emotional sounds as cues and auditory stimuli (Experiment 1) or visual stimuli (Experiment 2 and Experiment 3) as targets to explore whether auditory or visual spatial attention could be modulated by positive auditory cues. Experiment 3 also examined the temporal dynamics of cross-modal auditory bias toward positive natural sounds using event-related potentials (ERPs). The behavioral results of the three experiments consistently demonstrated that response times to targets were faster after positive auditory cues than they were after neutral auditory cues in the valid condition, indicating that healthy participants showed a selective auditory attentional bias (Experiment 1) and cross-modal attentional bias (Experiment 2 and Experiment 3) toward positive natural sounds. The results of Experiment 3 showed that N1 amplitudes were more negative after positive sounds than they were after neutral sounds, which further provided electrophysiological evidence that positive auditory information enhances attention at early stages in healthy adults. The results of the experiments performed in the present study suggest that humans exhibit an attentional bias toward positive natural sounds.

## Introduction

Emotional attention refers to the tendency to give preference to processing emotional information over neutral information (Vuilleumier, 2005; Yiend, 2010; Pourtois et al., 2013; Gerdes et al., 2020). The ability to detect and respond to threats in the natural world is of vital importance for adaptive behavior, survival across species (LeDoux, 1996; Silstona and Mobbs, 2018; Kreutzmann et al., 2020). Whereas danger signals predict the onset of a potentially threatening event, safety signals indicate its non-occurrence, thereby inhibiting fear and stress responses. Organisms evolved specific nervous systems (e.g., amygdala and hypothalamus), associative learning processes (e.g., Pavlovian conditioning) or cognitive mechanisms that rapidly direct attention to threatening information and keep our attention focused on a potential threat as long as needed. Since threat-related attentional processes have fundamental survival value (Mathews and Mackintosh, 1998; Mogg and Bradley, 1998), and studies on threat-related attentional processes will be very helpful to reveal the cause of anxiety disorders (Sheppes et al., 2013). Most studies that explore the attentional bias of emotional information have focused on the cognitive and neural mechanisms of attentional bias toward threatening stimuli or negative information through the indirect behavioral index (e.g., response times) or the direct physiological/neurological index (e.g., eye movement, event-related brain potentials) (Dong et al., 2017; Schmidtendorf et al., 2018; Berggren and Eimer, 2021). In addition, a large number of studies have investigated attentional bias toward negative information in healthy individuals as well as in participants experiencing a variety of anxiety disorders (Bar-Haim et al., 2007). Extensive research shows that negative attentional bias is closely related to the formation and maintenance of anxiety symptoms (Bar-Haim et al., 2007; Van Bockstaele et al., 2014; Ren et al., 2020; Salahub and Emrich, 2020; Basanovic et al., 2021). The components of negative attentional bias can be divided into two mechanisms: (a) initial attentional orientation toward orienting to threatening stimuli (engagement bias or attention capture); (b) difficulty disengaging attention from threatening stimuli (disengagement bias) (Posner et al., 1987; Cisler and Koster, 2010). Initial attentional orientation influences attention selection during early, automatic processing stages, which occur before 150 ms, and disengaging attention from stimuli occurs after the stimulus has been selected during late processing stages, which occur after 250 ms (Weierich et al., 2008; Pool et al., 2016; Wang et al., 2019b; Gupta et al., 2021; Yuan et al., 2021).

### Attentional bias for positive emotional stimuli

An increasing number of researchers have expanded the investigation into attentional biases toward positive emotional stimuli in healthy individuals (Wadlinger and Isaacowitz, 2008; Sali et al., 2014; Pool et al., 2016). Positive attentional bias means that individuals will preferentially pay more attention to positive stimuli or rewarding information than neutral stimuli (Brosch et al., 2008a; Wadlinger and Isaacowitz, 2008; Pool et al., 2016). Positive rewarding stimuli have been defined as stimuli that have a positive hedonic value that might elicit approach behaviors. Positive rewarding and negative threatening stimuli are both affectively relevant; positive attentional bias thus has very important evolutionary implications for an organism’s survival (Schultz, 2004; Anderson, 2016). If individuals prefer positive, rewarding information, they are more likely to recognize future benefits and have more positive beliefs and attitudes toward uncertainty, which can help them obtain survival resources or promote social status. Individuals experiencing positive attention biases may use selective attention as a tool to regulate their emotional experience during stressful circumstances and maintain a positive and stable mood, resulting in better mental health. Attention toward happy faces was positively correlated with positive mood and life satisfaction (Sanchez and Vazquez, 2014). A great deal of research has used different types of positive visual stimuli to explore positive attentional bias, including pictures of baby faces (Brosch et al., 2008a), happy faces (Joormann and Gotlib, 2007; Sanchez and Vazquez, 2014), pictures of food (Brignell et al., 2009; Tapper et al., 2010), positive words (Grafton and MacLeod, 2017), and stimuli features that are associated with reward outcomes (Anderson and Yantis, 2012, 2013; Pool et al., 2014; Sali et al., 2014). These studies consistently reported that, compared with neutral information, healthy individuals displayed attentional bias toward positive information. A recent meta-analysis systematically compared attentional biases for positive and neutral stimuli across 243 studies and showed that attention bias for positive stimuli among healthy individuals was larger in paradigms that measure early rather than late attentional processing, which suggests that positive attentional bias occurs rapidly and involuntarily (Pool et al., 2016). Moreover, it was also demonstrated that older adults exhibited attentional bias toward positive pictures at earlier stages in attention processing (Kennedy et al., 2020). However, another study investigated the time course of attentional bias toward positive visual stimuli (happy faces) using dot-probe paradigm, and revealed that happy faces were also associated with delayed disengagement during later stages of attentional processing (Torrence et al., 2017). Considering these inconsistent findings, it remains unclear at which stage positive emotion stimuli impacts attentional processing. Therefore, one of the purposes of the present study was to explore whether initial attention capture or subsequent disengagement was related to attentional bias toward positive information.

### Attentional bias toward emotional auditory information

Humans can accurately perceive the world and navigate in complex environments, which can contain several different emotionally relevant (or irrelevant) cues from different sensory modalities. Vision and audition represent two important senses needed to navigate through space and time (Bell et al., 2019; Choiniere et al., 2021; Peterson et al., 2022). It might be because the available, present-day technology is better suited for studying vision than for studying other modalities, there are more research on vision than on any other sensory modality (Hutmacher, 2019). Similarly, there were more studies on attentional bias toward visual emotional stimuli than on attentional bias toward emotional auditory information (e.g., Sheppes et al., 2013; Torrence et al., 2017). In fact, auditory sounds also convey emotionally relevant information and interact with vision to provide an appropriate judgment of the emotional qualities of a situation (Gerdes et al., 2014, 2020; Concina et al., 2019). Natural sounds in everyday life, such as screams, moans, cheers, applause, barks, and chirps, often contain emotional information and can carry biologically significant emotional information (Armony and LeDoux, 2010; Lepping et al., 2019; Wang et al., 2019b). For example, when we walk through busy streets, an alarm whistle can be a threatening cue that prompts an individual to make avoidance responses. Similarly, when we take a walk in the woods, the musical sounds made by birds can be rewarding signals and activate an organism to make approach-related behavior. If you hear the familiar call of friends, you will immediately look toward them with a smile. In other words, sounds serve as warnings or rewarding signals in daily life that can be critical for survival and fine-tune our actions (Harrison and Davies, 2013; Gerdes et al., 2020; McDougall et al., 2020). A recent study has investigated auditory attentional bias employing white noise as negative auditory cue (Wang et al., 2019a), but white noise lacks ecological validity. In fact, we need to detect and process natural sounds from real word more frequently in our everyday life (e.g., a barking sound of a dog or the sound of applause), resulting in avoidance-related or approach-related behaviors. Since natural sounds have been proved to be have good ecological validity (Hu et al., 2017; Mtynarski and McDermott, 2019), some researchers have suggested that studies that incorporate natural sounds should be encouraged (Sutherland and Mather, 2012; Koumura et al., 2019; Zhai et al., 2020; Zuk et al., 2020). Therefore, the present study aims to investigate whether participants exhibited attentional bias toward emotional natural sounds (positive and negative natural sounds).

### The cross-modal attentional bias toward emotional auditory information

In everyday life, precise processing of temporal and spatial information needs to consider cross-modal interactions in endogenous spatial attention between vision and audition. Selective attention has traditionally been studied separately for different sensory modalities, with little direct contact between traditional research on “visual attention” or “auditory attention.” The issue of cross-modal interactions in spatial attention between vision and audition has been addressed only more recently (Van Vleet and Robertson, 2006; Arieh and Marks, 2008; Blurton et al., 2015). Furthermore, recent studies have shown that negative auditory cues guide auditory or visual spatial allocation of attention (Peschard et al., 2017; Carlson et al., 2018; Wang et al., 2019a; Gerdes et al., 2020) or influence semantic processing (Gao et al., 2020). It has been found that emotional visual cues also modulate auditory spatial attention (Harrison and Woodhouse, 2016). This growing body of empirical evidence has proven that healthy participants exhibit auditory or cross-modal attentional bias toward negative auditory information. However, most studies have focused on whether or not positive visual stimuli modulated visual spatial attention (e.g., Pool et al., 2016), so it remains unclear whether positive natural sounds could modulate auditory or visual spatial allocation of attention. Therefore, the purpose of the present study was twofold: (a) to examine auditory and cross-modal attentional bias toward positive auditory information using natural sounds as positive cues; (b) to explore the cognitive and neural mechanisms underlying auditory and cross-modal positive attentional bias.

### The mechanism of attentional bias toward emotional information

Attentional bias toward negative information is thought to be caused by two components: facilitated engagement with negative information (involuntary attentional capture by negative stimuli) and delayed disengagement from negative information (volitional delayed disengagement from negative information) (Bar-Haim et al., 2007). The former component is called engagement bias, which refers to the quick detection of threat stimuli as opposed to non-threat stimuli (Cisler et al., 2009). The latter component is disengagement bias, which refers to the fact that it is harder to direct attention away from threatening stimuli (Fox et al., 2001, 2002). A threat detection mechanism likely underlies facilitated attention, reflecting an automatic processing. Delayed disengagement from negative information might be related to attention control abilities or emotion regulation goals, reflecting a top-down processing (Cisler and Koster, 2010; Park et al., 2013; Basanovic et al., 2021). Similarly, the components of positive attentional bias might also be linked to two factors: facilitated attention for positive information, which occurs at an early stage, and disengagement from reward information, which might occur at a later stage of attention processing (Pool et al., 2016). According to evolutionary theory, both attentional engagement with positive information and maintenance of attention with positive information underlie attentional bias for positive stimuli. Orienting to positive stimuli automatically helps an individual detect reward information in the environment rapidly, pursue potential survival resources, and improve social status (Strauss and Allen, 2006; Vuilleumier, 2015; Gupta, 2019; Gutiérrez-Cobo et al., 2019; Lockhofen et al., 2021). Delayed disengagement from positive information helps individuals keep their attention on positive stimuli to keep a positive and optimistic mood, accomplishing the goal of emotion regulation and mental health (Wadlinger and Isaacowitz, 2008; Demeyer and Raedt, 2013; Thoern et al., 2016; Booth and Sharma, 2020; Wadley et al., 2020). In the present study, we investigated these two possible explanations while comparing the early and later attentional mechanisms that underlie auditory/cross-modal attentional bias toward positive stimuli.

### The present study

To reveal the subcomponents of attentional bias toward emotional stimuli, researchers have created several experimental paradigms centered on classical cognitive tasks. These experimental paradigms included the dot-probe task (e.g., Yiend, 2010), visual search task (e.g., Wieser et al., 2018), free viewing task (e.g., Dong et al., 2017; Navalón et al., 2021), emotional Stroop task (e.g., Phaf and Kan, 2007; Kaiser et al., 2017), and emotional cueing task (e.g., Victeur et al., 2020). The emotional spatial cueing paradigm was modified from the exogenous cue-target task (Fox et al., 2001). Among these experimental paradigms, the emotional spatial cueing paradigm has been specifically designed to simultaneously measure initial orientation and difficulty with disengagement through a simple behavioral index (i.e., response latency) (Posner and Cohen, 1984; Pool et al., 2016; Preciado et al., 2017; Blicher and Reinholdt-Dunne, 2019; Wang et al., 2019a). However, recently, researchers have added a condition with two neutral cues furnishing a neutral baseline in the dot probe detection task (e.g., Carlson and Reinke, 2014), such modification becomes possible to measure initial orienting and later disengagement with the dot-probe task as well. Comparison of such a baseline with trials in which the target appears at the same location as the emotional cues reflects initial orienting, whereas comparison of the baseline with trials in which the target appears at the location opposite to the emotional cue reflects attention disengagement.

Some researchers also have proposed that using appropriate emotional stimuli and a behavior indicator could help reveal engagement bias and disengagement bias through the emotional spatial cueing paradigm (Fox et al., 2002; Schwerdtfeger and Derakshan, 2010; Imhoff et al., 2019; Wang et al., 2019a). In this task, participants direct their attention to a fixation point at the center of a screen. A cue (emotional or neutral) randomly appears on one side of the fixation point. Shortly after the cue offset, a target is presented either in the same location as the cue or on the opposite side. Participants are instructed to identify the location of the target as quickly and accurately as possible. Engagement bias is indicated through the facilitated detection of targets presented in the location occupied by an emotional cue (valid condition). Disengagement bias is indicated by slower responses to targets presented on the opposite side to an emotional cue (invalid condition) compared with responses to a neutral cue. Although the reliability of the emotional cueing paradigm has not been assessed, the reliability of other tasks that measure attentional bias toward threatening information (such as the dot-probe task and emotional Stoop task) was proven to be low (Van Bockstaele et al., 2014). Therefore, in this study, we also adopted the emotional cueing task to explore the subcomponents of attentional bias toward positive auditory stimuli in healthy individuals.

To date, although most studies on attentional biases have applied behavioral paradigms, event-related brain potentials (ERPs) are particularly suited for examining attentional biases. ERPs can provide a temporally precise, direct measure of emotional attention and may detect subcomponents of positive attentional bias that are not evident in behavioral data. Recently, a growing number of studies have adopted high temporal resolution event-related potential techniques (ERP) to examine the neural correlates of attentional bias toward emotional information (Keil et al., 2007; Hao et al., 2015; Reutter et al., 2017; Berggren and Eimer, 2021; Carlson, 2021; Schindler et al., 2021). These studies reported that some ERP components were involved in the neural mechanisms of attentional bias toward emotional negative information or threatening stimuli. Specifically, evidence showed that negative faces amplified the N170 ERP component (Schindler et al., 2021), evoked larger N2pc component (Berggren and Eimer, 2021), and increased late positive potential (LPP) (Schindler et al., 2021) in healthy individuals. Also, some studies revealed that higher (i.e., more negative) N2pc amplitude was found for angry faces in social anxiety (Reutter et al., 2017; Wieser et al., 2018) or disgust faces (Yuan et al., 2019). Using visual oddball task, researchers found that previous depression was uniquely associated with greater P3 ERP amplitude following sad targets, reflecting a selective attention bias toward negative faces (Bistricky et al., 2014). Some research groups have suggested that both healthy and anxious populations displayed modulations of early ERP components, including the P1, N170, and N2pc, in response to threatening and emotional stimuli, suggesting that both typical and abnormal patterns of attentional bias were characterized by enhanced allocation of attention to threat and emotion at earlier stages of processing, and modulations of later components, such as the P3, reflecting conscious and evaluative processing of threat and emotion and disengagement difficulties at later stages of processing (Torrence and Troup, 2018; Gupta et al., 2019; Carlson, 2021). However, few studies have revealed the dynamic time course of attentional bias for positive auditory stimuli in healthy adults. It remains unclear whether modulations of early ERP components or late ERP components were associated with attentional bias toward positive natural sounds in heathy adults. Because positive emotions and negative emotions have different evolutional functions for the survival of humans, it is important to examine the neural mechanism of attention bias for positive emotional information. To date, only one study has explored the exact time course of early attention allocation toward positive visual stimuli using a dot-probe paradigm in healthy participants, and it found that N1 amplitudes after positive pictures were enhanced compared to those that followed after negative pictures, indicating enhanced attention engagement with positive visual information (Pintzinger et al., 2017). In addition, one recent study revealed that the amplitude of the N1 component was enlarged by emotional sounds relative to that evoked by neutral sounds (Folyi et al., 2016). Another study revealed that patients with internet gaming disorder (IGD) exhibited increased attentional bias toward visual gaming-related cues; specifically, higher LPP amplitudes were found for game-related cues in the IGD group (Kim et al., 2018). Therefore, one goal of the present study was to investigate the neural mechanism of attentional bias toward positive natural sounds employing event-related potential techniques.

With the above in mind, in three experiments, we adopted emotional natural sounds as cues and auditory stimuli (Experiment 1) or visual stimuli (Experiment 2) as targets to explore auditory attentional bias (Experiment 1) or cross-modal attentional bias (Experiment 2) toward positive sounds using an auditory emotional cueing paradigm. Considering that the ERP approach is one method that can be used to better understand the time course of attentional bias (Torrence and Troup, 2018), Experiment 3 was conducted to investigate the neural mechanism of cross-modal attentional bias toward positive sounds in healthy participants using event-related potential techniques. Our expectation was that visual attention would be preferentially oriented toward positive sounds rather than neutral sounds. For the ERP results, we hypothesized that some earlier components fell on the electrophysiological index of enhancement attention by positive auditory stimuli, including N1, N2pc, etc. The present study will provide neural evidence for the time course of attentional bias toward positive auditory information.

## Experiment 1

### Method

#### Participants

Fifty-four right-handed undergraduate students (*N*_female_ = 42, 18–24 years, *M*_age_ = 20.73 years, *SD*_age_ = 2.91) were recruited for monetary compensation. A power analysis (G*Power, Version 3.1) (Faul et al., 2007) estimated that 36 participants would be needed to achieve a power of 0.95 (*f* = 0.25, α = 0.05, β = 0.95). We recruited a total of 58 participants, of which four participants were excluded from analyses for not appropriately following task instructions, resulting in a final sample of 54 participants. All participants had normal hearing and normal or corrected-to-normal vision. All participants reported no history of neurological or psychological disorders. The present study was approved by the local research ethics committee (HR 310-2019). Each participant signed an informed consent form before the experiment.

#### Materials

Ten positive sounds, ten negative sounds, and ten neutral sounds that differed in valence norms were selected from the expanded version of the International Affective Digitized Sounds system (IADS-E, Yang et al., 2018) and presented *via* headphones (see Appendix A). The sounds were determined by using the Self-Assessment Manikin (SAM; Bradley and Lang, 1994). The criteria for choosing the sounds consisted of pleasant valence (mean SAM valence norm score- > 6.00) and neutral valence (3.00 < mean SAM valence norm score- < 5.00) on a 1–9 scale. The sounds were from the natural environment and had high ecological validity (see Appendix A for details of the selected IADS-E sounds). In the IADS-E system, these sounds last 6000 ms (consisting of three segments of the same sound repetition with a length of 2000 ms each). A 2000-ms segment was selected from each original sound on the basis that it was representative of the emotional content of the original sounds. The materials were rated for valence and arousal by an independent group of participants (*n* = 49) using 9-point rating scales (valence: 1 = very unpleasant, 9 = very pleasant; arousal: 1 = not at all arousing, 9 = very arousing). The results showed that positive sounds (mean valence = 6.94 ± 0.75) were rated as more pleasant than the neutral sounds (mean valence = 4.84 ± 0.60), *t* (48) = 5.68, *p* < 0.001, and negative sounds (mean valence = 2.96 ± 0.57) were rated as more unpleasant than the neutral sounds (mean valence = 4.84 ± 0.60). There was no significant difference between positive sounds (mean arousal = 6.13 ± 0.44) and negative sounds (mean arousal = 6.22 ± 0.81) with respect to arousal ratings, *t* (48) = 1.02, *p* = 0.34. Both positive and negative sounds were rated as higher arousal than neutral sounds (mean arousal = 4.78 ± 0.62).

The auditory target stimulus was a 100 ms neutral tone (150 Hz, pure tone “beep”). The mean dB level for all sounds was 65 dB, measured at the participants’ ear. Auditory stimuli were presented randomly to either the left or right ear using EDIFIER W800BT headphones. E-Prime 2.0 was used to control the experiment.

#### Procedures

**FIGURE 1:**
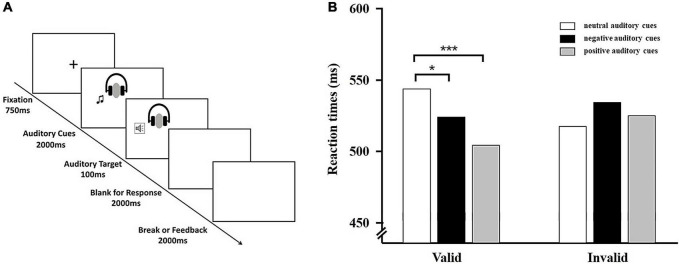
**(A)** Typical sequence of Experiment 1. Shown in the figure is an example of a valid trail. Emotional cues (positive, negative, or neutral) were presented equally often to the right or left ear and were followed by the target on the same (valid, 50%) or opposite side of space where the cue was presented (invalid, 50%). **(B)** Behavioral results from Experiment 1. Average reaction times for the six experimental conditions. Error bars indicate standard error (SE) of the mean. *p < 0.05; ***p < 0.001.

Participants were seated 60 cm from the screen and completed twenty practice trials first using auditory stimuli not included in the main experiment. Each trial began with a central fixation cross that lasted 750 ms. After the offset of fixation, an auditory cue (a positive sound, a negative sound or a neutral sound) was presented for 2000 ms. The sound duration was chosen to ensure that the emotional content of the sound was fully processed by the participants before the target appeared. Next, a neutral auditory target (pure tone) appeared for 100 ms. Participants were required to press the ‘F’ or ‘J’ key if the auditory target appeared on the left or right, respectively. If the auditory target appeared on the same side as the auditory cue, the auditory cue was regarded as valid; otherwise, the auditory cue was invalid. Participants had 2000 ms to respond after onset of the target. After response or after 2000 ms in the event of no response, an inter-trial interval of 2000 ms took place. All trials were presented in randomized order with an equal proportion of validly cued and invalidly cued trials (50%). In a validly cued trial, the target appeared on the side of the sound (positive/neutral); in an invalidly cued trial, it appeared on the opposite side of the sound (see Figure 1A).

We had a 3 (auditory cue valence: positive, negative vs. neutral) × 2 (cue validity: valid cues vs. invalid cues) within-subjects design. Each of these conditions was repeated 60 times, resulting in 360 trials. The experiment contained two blocks. Each block comprised 180 trials: 30 positive-valid trials, 30 positive-invalid trials, 30 neutral-valid trials, 30 neutral-invalid trials, 30 negative-valid trials, and 30 negative-invalid trials. Each block was separated by a short rest break. All stimuli were randomly assigned to each condition, and the assignment was counterbalanced between participants.

### Results

The mean error rate was 1.07%. These results were not analyzed further. Only reaction times on trials with correct responses were included in the analysis. Reaction times less than 150 ms and greater than 1500 ms were eliminated to eliminate premature responding to eliminate premature responding. RTs were submitted to a 3 (cue valence: positive vs. neutral vs. negative) × 2 (cue validity: valid cues vs. invalid cues) ANOVA. The main effect of cue validity was not significant, *F* (1, 53) = 0.14, *p* = 0.71, and there was no significant main effect of cue valence, *F* (2, 106) = 1.94, *p* = 0.15. Importantly, the crucial Cue Valence × Cue Validity interaction was significant, *F* (2, 106) = 12.74, *p* < 0.001, $\eta_{p}^{2}$ = 0.20. Further simple effect analysis of the two-way interaction revealed that RTs were shorter for positive auditory cues (*M* = 505.22 ms, *SD* = 101.66 ms) and negative auditory cues (*M* = 524.73 ms, *SD* = 103.59 ms) compared with neutral auditory cues (*M* = 544.31 ms, *SD* = 117.77 ms) in the valid condition. In the invalid condition, there was no significant difference between positive auditory cues (*M* = 525.57 ms, *SD* = 117.71 ms) and neutral auditory cues (*M* = 518.25 ms, *SD* = 109.89 ms), *t* (53) = 0.82, *p* = 0.42, negative auditory cues (*M* = 534.86 ms, *SD* = 105.49 ms) and neutral auditory cues (*M* = 518.25 ms, *SD* = 109.89 ms), *t* (53) = 1.58, *p* = 0.12 (see Figure 1B). For neutral cues, response times were slower in the valid condition (*M* = 544.31 ms, *SD* = 117.77 ms) relative to the invalid condition (*M* = 518.25 ms, *SD* = 109.89 ms), *t* (53) = 4.79, *p* < 0.001, which suggested that the IOR (inhibition of return) effect was observed. For positive cues, response times were faster to valid cues (*M* = 505.22 ms, *SD* = 101.66) relative to invalid cues (*M* = 525.57 ms, *SD* = 117.71), *t* (53) = −2.47, *p* < 0.05.

Then, we calculated the IOR effect by subtracting the mean reaction time on invalid trials from the mean reaction time on valid trials. This meant that a positive IOR index indicated a reluctance to return attention to the previously attended location, while a negative IOR index indicated a facilitation effect for the valid location and the absence of IOR. To compare the magnitude of IOR effect for different auditory cues, a repeated-measures was performed for the IOR index. This analysis showed a significant main effect of cue valence, *F* (2, 106) = 13.11, *p* < 0.001, $\eta_{p}^{2}$ = 0.38. Follow-up analyses of the main effect indicated that both of the IOR effects for positive auditory cues (*M* = −22.46 ms, *SD* = 8.29 ms) and the IOR effects for negative auditory cues (*M* = −7.54 ms, *SD* = 6.74 ms) were significantly smaller compared to neutral auditory cues (*M* = 26.06 ms, *SD* = 5.44 ms). Taken together, the IOR effect was reduced when cues were natural positive sounds, which suggested that participants displayed positive attentional bias toward auditory information from the natural environment.

### Discussion

Experiment 1 preliminarily explored attentional bias toward positive auditory stimuli from the natural environment in healthy participants and found that, like negative auditory sounds, positive auditory sounds also guide auditory spatial attention toward neutral sounds. The IOR effect was reduced more for positive auditory cues than neutral auditory cues. Furthermore, RTs were faster for positive natural sounds than for neutral sounds in valid condition. Given that the positive sounds are 2000 ms long, it is unclear whether these results indicate rapid capture, as there was a great deal of time after positive sound onsets during which engagement may begin. Therefore, we only concluded that healthy participants also displayed positive attentional bias toward positive natural sounds. In a recent study, researchers have revealed that healthy participants exhibited attentional bias toward aversive auditory stimuli (Wang et al., 2019a). The results of Experiment 1 further demonstrated the existence of attentional bias toward positive natural sounds in healthy individuals.

The above results are in agreement with previous studies on visual modal spatial cueing effects, which demonstrated that pleasant pictures influence the allocation of visual attention (Brosch et al., 2008a; Gable and Harmon-Jones, 2010; Peters et al., 2016; Pool et al., 2016; Kennedy et al., 2020). Our findings also provide evidence that confirms appraisal theories of emotion, which suggest that attention will be captured by stimuli that are relevant for the needs, goals, and well-being of the individual, irrespective of stimuli valence (Ellsworth and Scherer, 2003; Sander et al., 2005).

A cueing validity effect was found in emotional auditory conditions, while an inhibition of return effect (IOR) was found in the neutral auditory condition. Some research group has been investigated the neural correlates of attentional bias toward disgusting sound specifically in recent years (Zimmer et al., 2015, 2016). In their previous study, attention bias toward disgusting sounds was investigated using 1000 ms emotional sounds (aversive sounds vs. neutral sounds) as a cue, and it was found that an inter-stimulus interval (ISI) at 650–750 ms after neutral sound cues could induce IOR effects, but 1000 ms aversive sound cues plus an ISI at 50–750 ms was still found to induce cueing validity effects that prompted aversive sounds (Zimmer et al., 2019). Our results extended the previous findings that showed long SOA leads to IOR effects on neutral sounds, but the results of cueing validity effects on emotional sounds were still expanded to the longer stimulus onset asynchrony (SOA).

Experiment 1 investigated positive auditory bias in a single modality (auditory modality). However, in real-life environments, in addition to receiving the stimulation of a single sensory modality (such as the visual or the auditory channel), humans encounter simultaneous input from several different senses, such as vision and audition, when perceiving surrounding environmental changes, making appropriate behavioral responses, and coping with the complicated and changeable environment. Positive natural sounds also may carry biologically relevant emotional information that is useful to the mental and/or physical health of the individual. It remains unclear whether visual spatial attention is preferentially oriented toward positive natural sounds and how the involvement of each of the two components of attention bias subcomponent (engagement bias and disengagement bias) impacts cross-modal positive attention. Thus, Experiment 2 was conducted to investigate how positive sounds modulate visual spatial attention and aimed to further demonstrate the cross-modal attentional bias toward positive natural sounds using a modified audio-visuospatial cueing task.

## Experiment 2

### Method

#### Participants

Based on prior studies on the modulation of spatial attention to visual targets by auditory stimuli (Harrison and Davies, 2013; Wang et al., 2019a; Evans, 2020) and a power analysis using G*Power Version 3.1.9.2 (Faul et al., 2007) conducted for a repeated measurement ANOVA to detect a medium interaction effect of *f* = 0.25 with a statistical power of 0.95 and a significance level of 0.05, we aimed to recruit a sample size that included a minimum of 36 participants.

Sixty-one participants participated in Experiment 2 after giving informed consent. The results of two participants were excluded from analyses because they did not follow task instructions appropriately, resulting in a final sample of 59 participants (mean age = 20.81 ± 4.31 years, 13 male). All subjects were right-handed with normal hearing and normal or corrected-to-normal vision. All participants reported no history of neurological or psychiatric disorders. The experiment was approved by the Ethics Committee of East China Normal University (HR 310-2019).

### Materials

The emotional sounds materials were the same as those used in Experiment 1. The visual target was a white triangle (either downward or upward) presented on a black background at a 12-degree visual angle. The mean dB level for all sounds was 65 dB. Auditory stimuli were presented randomly to either the left or right ear over Sennheiser HD201 headphones.

### Procedures

**FIGURE 2:**
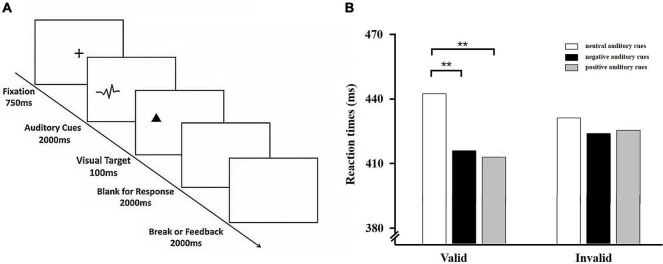
**(A)** Typical sequence of Experiment 2. Shown in the figure is an example of a valid trail. **(B)** Behavioral results from Experiment 2. Average reaction times to visual targets for the six experimental conditions. Error bars indicate standard error (SE) of the mean. **p < 0.01.

Participants were seated approximately 60 cm away from the monitor and introduced to the procedure. Each trial began with a fixation cross for 750 ms, followed by an auditory cue (positive, negative, or neutral) presentation that lasted for 2000 ms and appeared randomly in the left or right ear. Next, a visual target, a white triangle pointing upward or downward, appeared for 100 ms on the left or right side. During valid trials, the triangle appeared on the same side that was previously occupied by the emotional sound. During invalid trials, the triangle appeared on the opposite side of the auditory cue. Valid and invalid trials were presented in randomized order in equal proportions (50%). Participants were instructed to indicate the location (left or right) of the visual target (a white triangle) by pressing a key on the response keyboard (“F” or “J”) when the triangle was in a certain orientation (pointing upward or downward, counterbalanced across participants). Participants had 2000 ms to respond. Participants were also asked to respond as quickly and accurately as possible (see Figure 2A).

We used a 3 (auditory cue valence: positive vs. negative vs. neutral) × 2 (cue validity: valid cue vs. invalid cue) within-subjects design. The experiment contained two blocks. Each block included 180 trials: 30 positive-valid trials, 30 positive-invalid trials, 30 negative-valid trials, 30-negative-invalid trials, 30 neutral-valid trials, and 30 neutral-invalid trials. In total, the experiment consisted of 360 trials. After a practice phrase consisting of 20 trials, participants completed 360 experimental trials, of which 160 were valid trials and 160 invalid were trials. There was a 3 min break at the mid-point of the experimental trials.

### Results

The mean error rate was 0.99%. These results were not analyzed further. Incorrect trials and RTs less than 150 ms and greater than 1500 ms were eliminated from the RT analysis. Response times were analyzed in a 3 × 2 repeated measures ANOVA with the factors: cue valence (positive cue, neutral cue vs. negative cue) and cue validity (valid trials vs. invalid trials). Analysis revealed a significant main effect of cue valence, *F* (2, 116) = 4.92, *p* < 0.01, $\eta_{p}^{2}$ = 0.08, with faster reaction times for positive auditory cues (*M* = 415.13 ms, *SD* = 77.68 ms) and negative auditory cues (*M* = 415.77 ms, *SD* = 67.97 ms) than for neutral auditory cues (*M* = 430.34 ms, *SD* = 77.29 ms). The main effect of cue validity was not significant, *F* (1, 58) = 1.37, *p* > 05. Importantly, there was also a significant Cue Valence × Cue Validity interaction, *F* (2, 116) = 8.10, *p* < 0.01, $\eta_{p}^{2}$ = 0.12. Further simple effect analysis of the interaction effect revealed that RTs were faster for positive auditory cues (*M* = 409.78 ms, *SD* = 78.29 ms) and negative auditory cues (*M* = 412.32 ms, *SD* = 64.95 ms) than they were for neutral auditory cues (*M* = 435.17 ms, *SD* = 79.86 ms) in the valid condition, *F* (2, 116) = 8.10, *p* < 0.01, $\eta_{p}^{2}$ = 0.15. However, neither RTs for positive auditory cues nor RTs (*M* = 420.48 ms, *SD* = 77.06 ms) for negative auditory cues (*M* = 419.21 ms, *SD* = 70.99 ms) differed from RTs for neutral auditory cues (*M* = 425.51 ms, *SD* = 74.74 ms) in the invalid condition, *F* (2, 116) = 1.18, *p* > 0.05 (see Figure 2B).

For neutral auditory cues, response times were slower in the valid condition (*M* = 435.17 ms, *SD* = 79.86 ms) relative to the invalid condition (*M* = 425.51 ms, *SD* = 74.74 ms), *t* (58) = 2.13, *p* < 0.05, which suggested that the IOR effect was observed; however, for positive auditory cues, RTs were faster in the valid condition (*M* = 409.78 ms, *SD* = 78.29) relative to in the invalid condition (*M* = 420.78 ms, *SD* = 77.06 ms), *t* (58) = −2.92, *p* < 0.01, and also for negative cues, RTs were also faster in the valid condition (*M* = 412.32 ms, *SD* = 64.95 ms) than in the invalid condition (*M* = 419.21 ms, *SD* = 70.99 ms), *t* (58) = −2.17, *p* < 0.05.

Similar to Experiment 1, we also calculated the IOR effect by subtracting the mean reaction time on invalid trials from the mean reaction time on valid trials for different auditory cues. The one-way repeated measures for the magnitude of IOR effect revealed a significant main effect of cue valence, *F* (2, 116) = 8.10, *p* < 0.01, $\eta_{p}^{2}$ = 0.12. Follow-up analyses of the main effect indicated that both of the IOR effects for positive auditory cues (*M* = −10.70 ms, *SD* = 28.17 ms) and for negative auditory cues (*M* = −6.89 ms, *SD* = 24.43 ms) were significantly smaller compared to neutral auditory cues (*M* = 9.66 ms, *SD* = 34.86 ms). There was no significant difference in IOR effect between positive auditory cues and negative auditory cues. Taken together, the IOR effect was reduced when cues were emotional auditory cues relative to neutral auditory cues, which suggested that participants displayed cross-modal emotional attentional bias toward auditory information from the natural environment.

### Discussion

Experiment 2 further demonstrated that emotional auditory cues (both positive and negative natural sounds) can guide visual spatial allocation of attention. It also provided preliminary evidence that positive natural sounds modulate spatial attention to visual targets. The findings of Experiment 2 aligned with a recent study that examined spatial attention in 3-D space. It suggested that IOR size in the rewarded conditions was smaller than IOR size in the unrewarded condition and demonstrated that reward can attract much more attention in the near depth (Wang et al., 2022). Similarly, another study also demonstrated that social reward has the power to drive attention orienting behaviors (Hayward et al., 2018). Experiment 2 extended the findings of positive attentional bias that was found in auditory attention to the cross-modal domain. However, behavioral measures (reaction times) in Experiment 2 only provided an indirect measure of attentional processing. Event-related potentials (ERPs) are well-suited for investigating emotional attention bias because these measures allow for the examination of the time course of attention to emotional information with millisecond resolution (Kappenman et al., 2014, 2015). Researches on attentional bias toward visual emotional information have revealed that the modulations of early ERP components, including the P1, N170, and N2pc, suggesting that enhanced capture of attention by emotional stimuli at earlier stages of processing, and the modulations of later ERP components, such as the P3 or LPP components, indexing attention maintenance of emotion information at later stages of processing (Torrence and Troup, 2018; Gupta et al., 2019; Carlson, 2021). It remains unclear whether modulations of early ERP components or late ERP components were associated with attentional bias toward positive natural sounds in heathy adults. If individuals are sensitive to pursue potential survival resources or to obtain reward, healthy individuals tend to initially direct attentional resources toward positive natural sounds during early, automatic stages of processing adults, and might exhibit the modulations of earlier ERP components (e.g., N1, N2pc component). Or healthy adults tend to maintain their attention on positive stimuli to keep a positive and optimistic mood, accomplishing the goal of emotion regulation and mental health, indexing the modulations of later ERP components (e.g., P3 component). In the auditory domain, the cue-elicited N1 component was reported to be modulated by the emotional state at an early stage (Herrmann and Knight, 2001; Folyi et al., 2012). If the attention of emotional sounds is enhanced at the early stage, one should expect an increased amplitude of the N1 component evoked by emotional sounds relative to that evoked by neutral sounds (Folyi et al., 2016). P3 amplitude is thought to measure attentional allocation or attentional maintenance, and thereby the relative salience of a stimulus to a particular individual (Gasbarri et al., 2007; Bistricky et al., 2014). If greater attentional resources were allocated toward emotional sounds rather than neural sounds, we also expect greater P3 component evoked by emotional sounds. Thus, we conducted Experiment 3 to further explore the time course and neural substrates related to the processing of positive natural sounds using the event-related potential (ERP) technique in the same cross-modal emotional spatial cueing task used in Experiment 2. We expected that positive natural sounds would evoke an early attentional enhancement (e.g., the N1 component). Because the first two experiments demonstrated that healthy participants exhibited attention bias toward emotional sounds (both positive and negative), the purpose of Experiment 3 was undertaken mainly to investigate the neural mechanism of attentional bias toward positive natural sounds, so we only adopted positive and neutral natural sounds as auditory cues. We expected positive natural sounds could capture attention automatically, resulting in a greater N1 amplitude during earlier stages of attention processing in healthy individuals. In addition, in emotional spatial cueing task, positive auditory cues could facilitate attention to target detection in valid condition. Therefore, we also hypothesized that positive natural sounds would evoke greater P300 amplitude for validly cued targets relative to neutral natural sounds, which reflected conscious and evaluative processing or attention maintenance of positive natural sounds.

## Experiment 3

### Participants

The current sample size was chosen based on previous studies focused on the ERP correlates of attentional bias (e.g., Gupta et al., 2021; Woltering et al., 2021). A power analysis (G*Power, Version 3.1) (Faul et al., 2007) estimated that 36 participants would be needed to achieve a power of 0.95 (*f* = 0.25, α = 0.05, β = 0.95). Thirty-nine undergraduate students were recruited for monetary compensation. Three participants were excluded because of uncorrectable eye movement artifacts, and 36 subjects remained (*N*_female_ = 20, *M*_age_ = 21.10 years, *SD*_age_ = 2.16). All participants were right-handed and had normal or corrected-to-normal vision. None of the participants exhibited a history of psychiatric or neurological disorders. This study was approved by the Ethics Committee of East China Normal University (HR 310-2019), and all participants gave written informed consent.

### Experimental materials and procedure

The materials and procedure in Experiment 3 were similar to those used in Experiment 2 with the following exceptions. Negative sounds were not included in Experiment 3. Ten positive sounds and ten neutral sounds were adopted in the experimental materials, resulting in twenty sounds in total. All sounds were cropped to a duration of 2000 ms.

A within-subject design of 2 (auditory cue valence: positive auditory cues vs. neutral auditory cues) × 2 (cue validity: valid cues invalid cues) was used with 200 trials in each block (50 positive-valid trials; 50 positive-invalid trials; 50 neutral-valid trials; 50 neutral-invalid trials). The experiment contained two blocks. In total, the experiment consisted of 400 trials.

### EEG recording

Experiments were conducted in a dimly illuminated, anechoic, and sound-proof room. EEG was recorded continuously using Brain Vision Recorder, BrainAmp DC and a 64-channel actiCAP (Brain Products GmbH, Gilching, Germany) with a sampling rate of 1000 Hz and a FCz reference. The left and right mastoid electrodes were used as offline-reference. Electrodes placed at the outer right canthi measured the horizontal electrooculogram (HEOG), and electrodes below the left eye measured the vertical electrooculogram (VEOG). All electrode impedances were kept below 5 kΩ during data acquisition.

### Event-related potentials-data analysis

Signal processing and analysis were performed in MATLAB using EEGLAB toolbox version 14.1 (Delorme and Makeig, 2004). First, EEG data were registered to the standard BESA head mold, and the bilateral mastoid was reset as the reference electrode (TP9 and TP10). Data were filtered using a low-pass filter of 0.1 Hz and a high-pass filter of 30 Hz. Independent component analysis (ICA, Delorme et al., 2007) was performed on each participant’s data, and components that were clearly associated with eyeblinks or horizontal eye movements—as assessed by visual inspection of the waveforms and the scalp distributions of the components—were removed. Data exceeding ±80 μV were rejected, and remaining artifacts were manually removed, in total, 4.9% of the trials were excluded from further analyses. The remaining auditory cue-elicited epochs was segmented into epochs from 200 ms before and 2000 ms after the onset of auditory cue. Epochs were baseline corrected using the 200-ms pre-stimulus interval and averaged separately for the different valence conditions.

In the present study, electrode sites were chosen based on previous studies that focused on auditory spatial attention (Burra et al., 2019; Zimmer et al., 2019). We extracted the mean amplitude of the N1 component elicited by auditory cues, which was one of typical early auditory ERP components (Coch et al., 2005; Gädeke et al., 2013; Eddins et al., 2018; Topalidis et al., 2020; Rosburg and Mager, 2021) (in a cluster of six electrode sites (C1/C2, C3/C4, and T7/T8), covering the time from 130 to 200 ms after auditory cue onset). We also focused on the P300 component to visual targets in a cluster of five electrodes (CPz, CP1, CP2, CP3, and CP4) at a time window of 240–320 ms after the onset of visual targets.

### Results

#### Behavioral performance

The average accuracy for visual targets was 98.9%, *SD* = 0.92%. For the accuracy rates, a 2 (auditory cue valence: positive vs. neutral) × 2 (cue validity: valid cues vs. invalid cues) repeated measures ANOVA was conducted. No significant main effect or interaction was found (*F*s < 1, *p* > 0.05); therefore, these results were not analyzed further. Incorrect trials and RTs less than 150ms and greater than 1500 ms were eliminated from further analysis.

**FIGURE 3:**
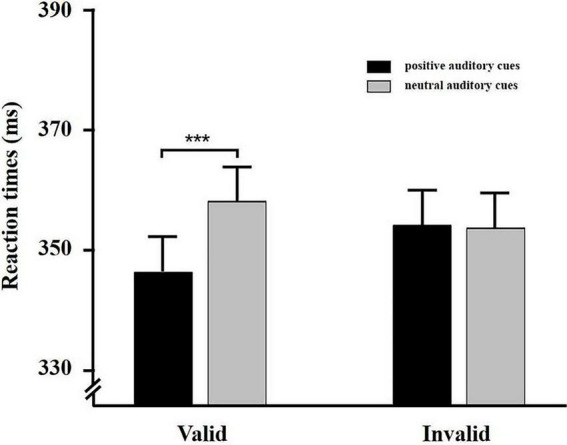
Behavioral results for Experiment 3. Average reaction times to visual targets for positive and neutral auditory cues. Error bars indicate standard error (SE) of the mean. ***p < 0.001.

A 2 (auditory cue valence: positive vs. neutral) × 2 (cue validity: valid cues vs. invalid cues) repeated measures ANOVA was performed for response times. The main effect of cue validity was not significant, *F* (1, 35) = 3.93, *p* > 0.05. The main effect of cue valence was also not significant, *F* (1, 35) = 0.96, *p* > 0.05 = 0.33. The Cue Valence × Cue Validity interaction was significant, *F* (1, 35) = 10.89, *p* < 0.01, $\eta_{p}^{2}$ = 0.24, suggesting faster responses for positive auditory cues (*M* = 345.92 ms, *SD* = 88.76 ms) relative to neutral auditory cues (*M* = 357.96 ms, *SD* = 93.34 ms) for valid trials, *t* (35) = −3.88, *p* < 0.001. No difference in RTs was observed between positive auditory cues (*M* = 353.89 ms, *SD* = 92.62 ms) and neutral cues (*M* = 353.38 ms, *SD* = 90.68 ms) for invalid trials, *t* (35) = −0.13, *p* > 0.05 = 0.89 (see Figure 3). We also calculated the IOR effect for different auditory cues. A *t*-test was conducted to compare the IOR effect difference between positive auditory cues and negative auditory cues. The analysis revealed that the IOR effect for positive auditory cues (*M* = −2.94 ms, *SD* = 18.58 ms) was significantly smaller than it was for neutral auditory cues (*M* = 4.47 ms, *SD* = 15.48 ms), *t* (35) = −2.15, *p* < 0.05. These findings were replicated with the results of Experiment 2, which revealed that healthy participants exhibited enhanced visual spatial attention to positive auditory cues.

#### EEG results

Based on previous studies (Keil et al., 2014; Zimmer et al., 2015; Cai et al., 2020), we focused on the N2ac brain wave components (C3/4, CP5/CP6, FC5/FC6, and T7/T8 electrodes) and N1 (C1/C2, C3/C4, and T7/T8 electrodes) components evoked by emotional sound cues, and the calculation method of N2ac was contralateral wave minus ipsilateral wave deaveraging at left and right brain electrodes. A time window of 200–300 ms after the onset of auditory stimulation was selected for the N2ac component (Burra et al., 2019), and a time window of 130–200 ms was selected for the N1 component (Zimmer et al., 2015; Burra et al., 2019). However, we did not observe the N2ac component in the present experiment. This is most likely due to our paradigm. An N2ac component is observed when cue and target are in close proximity. It is absent when stimuli are presented one at a time, as they were in the present study (Gamble and Luck, 2011; Luck, 2012, for a review; Zimmer et al., 2015). We also focused on the P300 component to visual targets in a cluster of five electrodes (CPz, CP1, CP2, CP3, and CP4) at a time window of 240–320 ms after the onset of visual targets.

### N1 amplitude in response to auditory cues

**FIGURE 4:**
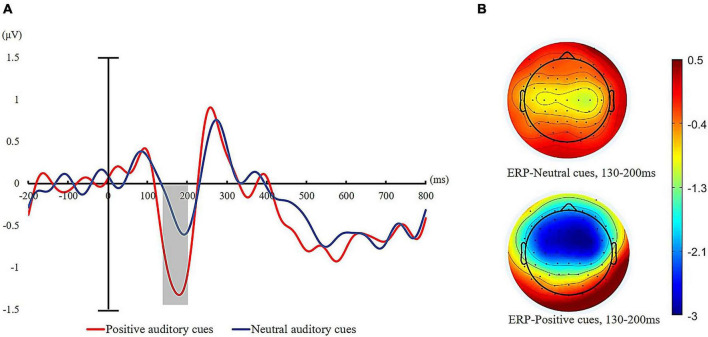
**(A)** ERPs recorded to the different emotional auditory cues (neutral = blue line and positive = red line) averaged across all participants. **(B)** Topographic maps for the different auditory cues at the mean peak of the N1 component (130–165 ms after the onset of auditory cues).

We calculated the averaged voltages for each type of auditory cue corresponding to a time window of 130–200 ms on N1 amplitude. For the N1 component elicited by auditory cues, a 2 (cue valence: positive auditory cue, neutral auditory cue) × 2 (hemispheric laterality: contralateral vs. ipsilateral) repeated-measure ANOVA was conducted. Analysis of the mean N1 amplitudes following the onset of auditory cues revealed a significant main effect of cue valence, *F* (1, 35) = 78.15, *p* < 0.001, $\eta_{p}^{2}$ = 0.69. Follow-up analysis of the main effect revealed that the average N1 amplitude elicited by auditory cues was significantly more negative for positive sound (*M* = −1.12 μV, *SD* = 0.65 μV) relative to neutral sound (*M* = −0.33 μV, *SD* = 0.52 μV). The grand average N1 waves for the two auditory cues are displayed in Figure 4. There was also a significant main effect of hemispheric laterality, *F* (1, 35) = 13.76, *p* < 0.01, $\eta_{p}^{2}$ = 0.26. The N1 amplitude was significantly larger over the hemisphere contralateral to the sound location than the ipsilateral hemisphere (contralateral: *M* = −0.83 μV, *SD* = 0.72 μV; ipsilateral: *M* = −0.44 μV, *SD* = 0.68 μV). The interaction for cue valence × cue validity was not significant, *F* (35) = 0.35, *p* > 0.05.

### P300 amplitude in response to visual targets

**FIGURE 5:**
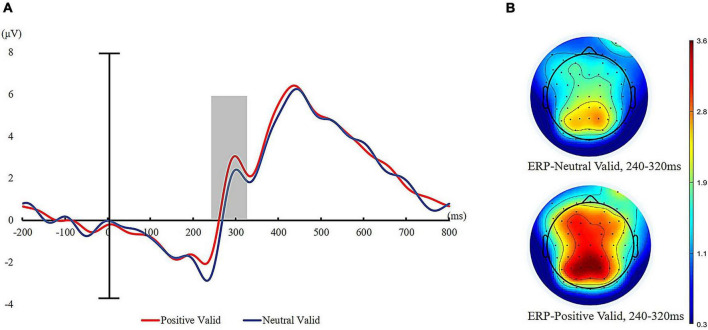
ERPs time-locked to visual target presentation for valid trials. **(A)** Grand average waveforms of P300 to valid targets at five sites (CPz, CP1, CP2, CP3, and CP4) for positive auditory cues and neutral auditory cues (positive valid = red line and neutral valid = blue line). **(B)** Topographic maps to visual targets presentation (240–320 ms after the onset of visual targets).

We also calculated the mean amplitudes of P300 after the onset of visual targets at centroparietal sites (CPz, CP1, CP2, CP3, and CP4). Repeated measures ANOVAs of 2 (cue valence: positive vs. neutral) × 2 (cue validity: valid cues vs. invalid cues) on mean amplitude of P300 revealed a significant main effect of cue valence, *F* (1, 35) = 12.99, *p* < 0.01, $\eta_{p}^{2}$ = 0.27, such that visual targets elicited greater P300 amplitudes for positive auditory cues (*M* = 2.50 μV, *SD* = 1.89 μV) than neutral auditory cues (*M* = 1.95 μV, *SD* = 1.51 μV). The main effect of cue validity was also significant, with the P300 amplitude significantly higher for invalid trials (*M* = 2.58 μV, *SD* = 2.71) than for valid trials (*M* = 1.88 μV, *SD* = 1.87 μV), *F* (1, 35) = 11.22, *p* < 0.001, $\eta_{p}^{2}$ = 0.24. The interaction of cue validity and cue valence was also significant, *F* (1, 35) = 4.44, *p* < 0.05, $\eta_{p}^{2}$ = 0.11. Further simple effect analysis showed that valid targets evoked a larger P300 for positive auditory cues compared to neutral auditory cues (positive: *M* = 2.29 μV, *SD* = 1.98 μV vs. neutral: *M* = 1.47 μV, *SD* = 1.75 μV), *t* (35) = 5.06, *p* < 0.001. However, the mean amplitude of P300 evoked by invalid targets was not statistically distinguishable between positive auditory cues (*M* = 2.71 μV, *SD* = 1.81 μV) and neutral auditory cues (*M* = 2.43 μV, *SD* = 1.61 μV) (see Figure 5).

### Discussion

Experiment 3 replicated and extended the results of Experiment 2. In Experiment 3, behavioral results again found that healthy individuals exhibited attentional bias toward positive auditory emotional stimuli, as indicated by faster responses to positive auditory cues than neutral auditory cues in the valid condition. Furthermore, the ERP results of Experiment 3 found an increased amplitude of N1 triggered by positive auditory cues relative to that triggered by neutral auditory cues, suggesting that enhanced attention with positive natural sounds compared with neutral natural sounds at a perceptual stage of auditory processing. In addition, we found that the amplitude of P300 evoked by visual targets was significantly larger for positive auditory cues than neutral auditory cues on valid trials, illustrating that those positive emotional sounds increased attention allocation to subsequent same-location visual target stimuli. Therefore, the converging behavioral and electrophysiological evidence of Experiment 3 suggested that healthy individuals exhibited cross-modal attentional bias toward positive natural sounds, and this positive auditory attentional bias was related to facilitated attentional engagement with positive natural sounds.

## General discussion

Across three experiments that combined behavioral and event-related potential techniques, the present study investigated auditory attentional bias (Experiment 1, auditory spatial cueing task) and cross-modal attentional bias (Experiment 2 and Experiment 3, cross-modal spatial cueing task) toward positive sounds from natural environment and further revealed the neural time course of attentional bias toward positive auditory information (Experiment 3). The behavioral results of these three experiments consistently demonstrated that positive auditory cues can modulate auditory/visual spatial allocation of attention. The electrophysiological findings of Experiment 3 suggest that attentional bias toward positive natural sounds occurs rapidly and during early stages of attention processing, which reflected the amplitude of N1component. This result aligns with the view that processing of emotional stimuli can be facilitated by rapid attention (Yiend, 2010; Folyi et al., 2016; Pool et al., 2016). In addition, the results of Experiment 3 also showed that the amplitudes of P300 component was enlarged for valid targets when cued by positive sounds than by neutral sounds, which might indicate positive natural sounds can also facilitate conscious attention maintenance of positive information at later stages of attention processing.

Attentional bias for emotional stimuli has attracted considerable interest in neuroscience (Vuilleumier, 2005; Yuan et al., 2019) and psychology (Yiend, 2010; Van Bockstaele et al., 2014). Initially, experimental research in both fields mainly focused on negative attentional bias in healthy individuals (e.g., Preciado et al., 2017) or anxiety individuals (e.g., Sheppes et al., 2013). The preferential allocation of selective attention to threatening stimuli can promote the efficient detection of potential dangers in the environment (Öhman and Mineka, 2001; Pourtois et al., 2013). However, attentional bias toward positive stimuli also plays an important role in human reproduction, maintaining mental health, and coping with stress (Dandeneau et al., 2007; Moors, 2014; Anderson, 2016; Thoern et al., 2016). Until recently, most studies that examine the impact of positive emotional stimuli on spatial attention have focused on the visual domain (Brosch et al., 2008b; Gable and Harmon-Jones, 2010). In addition to having spatial or physical characteristics, sounds can also transport object-specific information that may help individuals identify or detect visual objects. The acoustic channel is particularly well suited for signifying warning signals (Haas and van Erp, 2014). Therefore, we conducted the present study with the aim of exploring how positive natural sounds modulate auditory and visual spatial attention.

The behavioral results of these three experiments consistently revealed that response times were faster for positive auditory cues than for neutral auditory cues in the valid condition, and the magnitude of IOR was reduced in positive trials relative to neutral trials. Our findings confirmed that positive natural sounds were preferentially processed over neutral auditory information and guide auditory or visual spatial attention. These results aligned with previous findings on negative auditory attentional bias (Asutay and Västfjäll, 2015; Wang et al., 2019a,b; Bonmassar et al., 2020) or negative visual attention bias (Harrison and Davies, 2013; Gerdes et al., 2014, 2020; Burra et al., 2019). In the first two experiments, we also found that participants exhibited a similar response pattern to positive and negative sounds; in other words, participants showed attentional bias toward emotional sounds. Therefore, attentional bias for these two kinds of emotional stimuli (positive rewarding and negative threatening stimuli) should be similar (Pool et al., 2016). The present study provided initial evidence suggesting that humans exhibited an attentional bias toward positive natural sounds. Similar to positive visual information, positive natural sounds (e.g., chirpings of birds; sounds of gurgling water) can also carry significant emotional information (such as potential survival resources or opportunities, indicating novelty and diversity in nature), resulting in faster responses to auditory/visual targets.

Behavioral measures of attentional bias toward positive sounds failed to clarify the time course of the positive attentional bias. Therefore, Experiment 3 explored the ERP component of positive attentional bias and found that positive natural sounds elicited larger N1 amplitudes (130–200 ms after emotional auditory cues onset) relative to neutral sounds, suggesting an involuntary orienting of attention toward positive auditory cues. Recent reviews on the neural correlates of threat-related attentional bias have shown that both healthy and anxious populations exhibited facilitated engagement with negative stimuli, as shown by the greater amplitude of early ERP components, including the N1 component (Gupta et al., 2019). Previous studies that focused on auditory attention have shown that the increased amplitude of N1 component was generally considered to be an increase in attentional resources to acoustic cue stimuli (Hillyard et al., 1973; Lange et al., 2003; Doricchi et al., 2020). Based on previous findings on the N1 component, the present study indicated that the modulation of positive sounds on spatial attention emerged at quite early stages of attention.

Our results aligned with the findings of previous studies on the neural mechanism of attentional bias toward emotional visual information (Santesso et al., 2008; Pintzinger et al., 2017). It also has been shown that the N1 amplitude is larger for both pleasant and unpleasant images compared to neutral images (Olofsson et al., 2008; Foti et al., 2009; Grassini et al., 2019) and for cannabis-related cues in cannabis use disorders (Ruglass et al., 2019). Moreover, some studies that focused on auditory attention have shown that the increased amplitude of N1 component was generally considered to signify enhanced attention engagement with auditory cues (Hillyard et al., 1973; Woldorff et al., 1987; Lange et al., 2003; Doricchi et al., 2020). Evidence showed that the amplitude of the N1 component was enlarged for infant high-distress cries in mothers (Maupin et al., 2019), for task-relevant tones (Alho et al., 1994; Lange, 2013), and in the difficult auditory detection task (Sabri et al., 2006). Thus, the enlarged N1 amplitude in Experiment 3 might reflect enhancement of auditory attention captured by positive auditory stimuli in normal populations, which aligns with a recent study focused on attentional bias toward positive visual information (Pintzinger et al., 2017). Further, these results suggested that healthy adults preferred to process rewarding stimuli involuntarily within the auditory field. The findings of the present study are also in agreement with an extensive body of literature on multi-modal spatial cueing effects, which demonstrates that cues in one sensory channel can guide spatial attention allocation in another modality (Zimmer et al., 2015; Pierce et al., 2018; Gerdes et al., 2020).

In Experiment 3, visual targets elicited larger P300 amplitudes for positive auditory cues compared to neutral auditory cues on valid trials. Previous studies have shown that the amplitude of P300 was related to attentional resource allocation to emotional stimuli (Yee and Miller, 1994; Kessels et al., 2010; Willner et al., 2015), attentional disengagement with threat (Baik et al., 2018), the maintenance of attention (Bauer, 2021). Experiment 3 found that P300 amplitudes were higher for visual targets after positive auditory cues than those for visual targets after neutral auditory cues in valid trials, but not in invalid trials. Therefore, it is reasonable to assume that positive auditory cues facilitated attention resource allocation to visual targets preceded by positive auditory cues. Taken together, the findings of Experiment 3 revealed electrophysiological evidence that attention allocation to positive auditory information occurred at earlier stages in healthy adults, which was consistent with previous findings (Nelson and Hajcak, 2017; Ferry and Nelson, 2021). More importantly, in a recent meta-analytic study, researchers systematically compared attentional bias toward positive visual stimuli across 243 studies, and they suggested that attentional bias for positive visual information occurred rapidly and involuntarily during early stages of attention processing (Pool et al., 2016). To our knowledge, this is the first study to provide converging evidence that positive auditory information enhances both initial and sustained attention in healthy individuals. We also expanded the previous studies conducted on visual stimuli to auditory stimuli of natural word. This may help to transfer our findings of cross-modal influences on visual attention to more natural settings.

There are a few limitations. First, effect sizes for observed experimental effects are relatively small, one explanation is that the number of trials is low. Future studies should use a larger number of trails to examine this question. Another possible explanation is the effect size of attentional bias toward positive information was rather small. One recent meta-analysis showed that healthy individuals existed reward-related attention distraction with a small effect size (Rusz et al., 2020). Another meta-analytic investigation has reported that the effect size of the positive attentional bias using spatial cueing task was smaller compared with rapid serial visual presentation paradigm (Pool et al., 2016). Second, we did not include negative sounds in Experiment 3. It would be helpful to compare the ERP correlates of negative with those of positive sounds. Nonetheless, we emphasize that the main goal of the current study is on the differential attentional bias toward positive natural sound vs. the neutral sound distinction and how these effects are influenced in auditory and cross-modal setting.

Future studies can be refined in the following aspects. First, according to circumplex theories of emotion, all emotions are underlain by two orthogonal dimensions: valence and arousal (Russell, 1980; Anderson, 2005). Based on this assumption, positive emotional stimuli influence attentional selection contingent on their potential to elicit emotional arousal. Thus, a potential direction for future research involves exploring whether the size of positive attentional bias could be predicted by different arousing positive natural sounds. Second, only healthy adults were included in the present study. It may be extremely useful to examine the cognitive and neural mechanism of attention bias toward positive auditory emotional stimuli in clinical samples (e.g., depression, anxiety and dysphoric individuals), because doing so could help us understand the mechanisms that underline the etiology and maintenance of emotion disorders (Elgersma et al., 2018; Sadek et al., 2020). Third, emotion appraisal theory proposes that the psychological mechanism that drives emotional attention is the detection of a stimulus that is relevant to the observer’s concerns and goals (Sander et al., 2005). Therefore, future studies need to manipulate the stimulus to make it relevant for the participants’ concern in order to examine attentional bias toward positive auditory stimuli. For example, the sound of laughter from intimate friends has a much higher level of relevance to the participant than the laughter of strangers, resulting a larger attentional bias. Fourth, the present study only investigated the modulational effect of positive environmental sounds on subsequent neutral visual or auditory target stimuli. In order to comprehensively investigate cross-modal attention bias toward positive emotional stimuli, further studies are needed to specifically investigate whether positive visual cues (or simultaneously presented auditory cues) might modulate auditory attention. Finally, future investigations are also needed to develop a more nuanced understanding of how these effects generalize across discrete emotions (e.g., disgust, sadness, joy, fear, etc.) rather than just comparison general positive/negative understanding as of now.

## Conclusion

In summary, to our best knowledge, the present study is the first to investigate the mechanism of attentional bias toward positive natural sounds in healthy individuals. In three experiments, both behavioral and electrophysiological data converge on the central finding that normal individuals exhibited attention enhancement with positive auditory emotional stimuli, and the results of the N1 component in Experiment 3 provide direct electrophysiological evidence that attention bias toward positive auditory stimuli occurs very rapidly, involuntarily, and during the early stages of the attentional process. In general, our results aligned with evidence that there is an attentional bias toward positive visual information and further demonstrated that positive auditory information is processed preferentially.

## Data availability statement

The original data presented in the study are included in the article/supplementary material, further inquiries can be directed to the corresponding author/s.

## Ethics statement

The studies involving human participants were reviewed and approved by the Ethics Committee of East China Normal University (HR 310-2019). The patients/participants provided their written informed consent to participate in this study.

## Author contributions

YW and ZT conceived of the project, designed the experiments, implemented the experiments, and collected the data. YW, ZT, XZ, and LY wrote the manuscript. All authors approved it for publication.

## Appendix

**TABLE A1:** List of emotional auditory al sounds used in the current study.

Number	Description	Valence
206	shower	neutral
262	yawn	neutral
278	walking	neutral
318	sound of zipper	neutral
372	wing shaking	neutral
442	temple bell	neutral
483	clock	neutral
730	typing	neutral
829	page turning	neutral
922	washing clothes	neutral
111	music box	positive
123	sea	positive
151	robin	positive
172	brook	positive
200	valley	positive
298	gurgling water	positive
351	applause	positive
419	bird song	positive
1038	gurgling water	positive
1385	baby laugh	positive
255	vomit	negative
273	crash	negative
276	scream	negative
292	male scream	negative
293	bomb	negative
296	women crying	negative
432	shooting1	negative
434	shooting2	negative
602	thunderstorm	negative
626	explosion	negative
